# Firearms, storage practices, and child blood lead levels and household dust lead loadings: findings from the HOME study

**DOI:** 10.1038/s41370-026-00928-w

**Published:** 2026-05-30

**Authors:** Christian Hoover, Yingying Xu, Bruce Lanphear, Kimberly Yolton, Nicholas Newman, Joseph M. Braun

**Affiliations:** 1 Department of Epidemiology, Brown School of Public Health, Providence, RI, USA.; 2 Harvard Injury Control Research Center, Harvard T.H. Chan School of Public Health, Boston, MA, USA.; 3 Department of Pediatrics, Cincinnati Children’s Hospital Medical Center, University of Cincinnati College of Medicine, Cincinnati, OH, USA.; 4 Faculty of Health Sciences, Simon Fraser University, Burnaby, BC, Canada.; 5 BC Children’s Hospital Research Institute, Simon Fraser University, Burnaby, BC, Canada.

**Keywords:** Metals, Children’s Health, Early Life Exposure, Indoor dust/house dust/dust, Exposure Assessment, Biomonitoring

## Abstract

**BACKGROUND:**

Lead is a persistent environmental toxicant associated with adverse neurodevelopmental and behavioral outcomes, with children particularly vulnerable due to heightened exposure and biological susceptibility. Although population blood lead levels in U.S. children have declined substantially, a large proportion remain above levels associated with harm, indicating ongoing exposure from additional sources. Firearms use lead-based ammunition and primers and discharging a firearm releases lead particles that can be inhaled or transported into residential environments via take-home contamination.

**OBJECTIVE:**

To examine whether firearm ownership and storage practices were prospectively associated with children’s blood lead levels and residential dust lead.

**METHODS:**

In the HOME Study (Cincinnati, Ohio, enrolled 2003–2006), blood lead levels (μg/L; 10 μg/L = 1 μg/dL) were measured at ages 12, 24, and 36 months (*n* = 323) and floor dust lead (μg/ft^2^) at baseline, 12, and 24 months (*n* = 316). Caregivers reported firearm ownership, number of firearms, and storage practices during pregnancy. We fit generalized estimating equations to log_2_-transformed blood lead levels and log_10_-transformed dust lead, adjusting for income, race/ethnicity, flooring, cleanliness, maternal age, and child sex.

**RESULTS:**

Among firearm owners (*n* = 57–59 per analysis), homes where firearms were not stored or locked had 29–84% higher dust lead at baseline through age 24 months and children had ~20% higher blood lead levels at ages 12–36 months. Associations between firearm ownership and dust and blood lead levels appeared mostly null.

**SIGNIFICANCE:**

Among firearm owners, less safe storage was associated with higher floor dust and child blood lead levels, though some results were imprecise. Safe firearm and ammunition storage may reduce pediatric lead exposure in gun-owning households.

**IMPACT:**

- This prospective birth cohort study evaluates household firearms and storage practices as an understudied source of pediatric exposure. Using repeated measures of children’s blood and residential floor dust lead concentrations, we examined whether firearm ownership and firearm/ammunition storage practices during pregnancy were associated with lead biomarkers from birth through 36 months. Findings suggest that, among firearm-owning households, less secure storage practices are associated with higher residential dust lead and higher child blood lead levels. These results highlight a potentially modifiable exposure pathway relevant to environmental exposure science and pediatric lead prevention.

## INTRODUCTION

Lead is a toxic metal associated with cognitive impairment, cardiovascular disease, aggression, and impulsivity [1-3]. Children are more susceptible to lead than adults due to developmentally appropriate behaviors (e.g., hand-to-mouth) and sensitive to its effects given their developing nervous system [4]. The World Health Organization considers ≥50 micrograms per liter (μg/L) in blood to be elevated while the US Centers for Disease Control and Prevention (CDC) has a lower threshold, ≥35 μg/L [5, 6]. The CDC revised this threshold down twice between 2010 and 2025, which influenced our decision to report blood lead levels in μg/L rather than the more common microgram per deciliter (μg/dL; 10 μg/L = 1 μg/dL) [5, 7]. While geometric mean (GM) blood lead levels in United States (U.S.) children decreased from 150 μg/L between 1976-1980 to 8.3 μg/L by 2016, measurable exposure remains common [8, 9]. In a national clinical laboratory study of 1.1 million U.S. children, more than half of those tested had blood lead levels ≥10 μg/L [9]. This is concerning given that elevated blood lead levels are associated with internalizing symptoms, aggression, impulsivity, and violent injuries such as firearm-related suicide or victimization [10-12]. Historically, the dominant sources of lead include house dust contaminated with lead-based paint, drinking water from lead-based pipes, and air contaminated with leaded gasoline exhaust [13]. While these sources have been or continue to be mitigated through successful public health programs, additional sources of lead such as caregiver occupational take-home exposure, lead contaminated food, and hobbies of family members could place children at risk of exposure and associated adverse health effects [14].

Firearms and ammunition can be a source of lead exposure in children. Firearms use lead-based ammunition and primers and, as evinced by ecology and occupational studies, lead-based ammunition can increase blood lead levels [15-17]. Research has shown that the lead contained in bullets and ammunition is expelled from the firearm into the air and can be inhaled by the shooter or bystanders [16, 18]. Among recreational shooters, median blood lead concentrations ranged from 33 μg/L to 1075 μg/L and increased by caliber of firearm [16, 19]. Secondary and tertiary exposures can occur through lead settling on clothes and personal effects in the environment, which can then migrate onto shoes or clothes and be deposited in cars and in homes (i.e., take-home lead exposure) [16, 20].

Ecological and cross-sectional studies have found that children living in U.S. States and cities/towns with more firearm licensure have higher blood lead levels than their peers in lower licensure communities [21-23]. For example, children living in communities with the highest quartile of firearm licensure between 2010-2019 had 18% higher rates of elevated blood lead levels relative to the lowest quartile [22]. Among U.S. States between 2012 and 2018, each interquartile range (IQR) increase in household firearm ownership (14%) was associated with a 41% increase in the prevalence of children with blood lead levels above 50 μg/L [23]. However, we are unaware of any studies examining the effect of firearm ownership on residential dust lead loadings and blood lead levels of children at the individual level. To address this gap, we used data from a prospective birth cohort to examine the relationship of self-reported firearm ownership and storage behaviors with serial floor dust lead loadings and childhood blood lead levels from birth to age 36 months.

## METHODS

### Study participants

We collected data from mother-child dyads participating in the Health Outcomes and Measures of the Environment (HOME) Study in Cincinnati, Ohio. The HOME Study is a prospective pregnancy and birth cohort that followed 420 children from 2nd trimester to early adulthood to assess the effects of environmental toxicant exposures on various health outcomes [24, 25]. Of 420 children actively followed in the cohort, 323 had data on firearm ownership, at least one blood lead concentration at ages 12, 24, or 36 months, and complete covariate data; 316 had dust lead data at baseline, 12 months, and 24 months (Table 1). Eligibility was determined via clinic records and phone interviews and included adult, English-speaking, HIV-negative pregnant women ( < 19 weeks) living in pre-1978 (national ban on lead in paint) homes in the greater Cincinnati area, without some major chronic or psychiatric condition (e.g., diabetes, bipolar), and planning to continue prenatal care, deliver locally, and remain in the area for at least one year. To enrich for lead exposure risk and examine disparities, enrollment was stratified by geography and oversampled women who self-identified as Black [24].

### Firearm ownership and storage behaviors

Firearm ownership was assessed through four self-reported questions administered to the caregiver during a baseline study visit at approximately 20 weeks’ gestation: (1) “Is there a firearm in this home?” (yes vs. no); (2) “How many firearms are there in the home?” (0, 1, 2 + ); (3) “Are all firearms kept separate from the ammunition?” (no firearms, ammunition stored separately, not stored separately); and (4) “Are all firearms in a locked box and fitted with a trigger lock?” (no firearms, all firearms stored/locked, not all firearms stored/locked). Because firearm storage practices can only be meaningfully assessed among those who own firearms, and because of demographic differences between firearm owners and non-owners, we ran two sets of models for each outcome: one among all participants and one among firearm owners only [26, 27].

### Blood lead concentrations

We collected whole venous blood at ages 12, 24, and 36 months using lead-free materials. Blood samples were stored at −80 °C and shipped to CDC laboratories on dry ice where lead concentrations were quantified using inductively coupled plasma mass spectrometry methods [28]. All batches included reagent blanks and quality control samples, with QC sample coefficient of variation <3.5%.

### Dust lead loadings

We measured dust lead loadings (μg/ft^2^) in floor dust collected at the baseline study visit and again at months 12 and 24. At each visit, we collected dust from floors (1–sq ft area) from the kitchen, main activity room, and child’s bedroom using wipes that were lot-tested for lead contamination. We collected three samples per surface and combined them into a single composite sample. Efforts were made to sample the same area of floor across all time points. Dust lead loadings were quantified using flame atomic absorption spectrometry (FAAS), graphite furnace atomic absorption spectrometry (GFAAS), and inductively coupled plasma spectrometry (ICP-MS) following established laboratory procedures [24, 29-31]. The method detection limit (MDL) for FAAS, GFAAS, and ICP-MS were approximately 1–2 μg per sample, 0.01–0.2 μg per sample, and ~0.4 μg per sample, respectively [30, 31]. Baseline dust used FAAS and GFAAS when samples were <5 μg, with GFAAS values selected for reporting/analysis. Dust samples at months 12 and 24 were analyzed with ICP-MS. Dust lead values below the MDL were assigned a $\frac{LOD}{\sqrt{2}}$ at the sample level and subsequently expressed as μg/ft^2^.

### Covariates

We identified variables that could confound associations of firearm presence with dust lead loadings or blood lead concentration using a directed-acyclic graph (DAG) (Supplementary Fig 1). For dust lead, we adjusted for household income (fitted with a cubic spline term), race/ethnicity, carpet level, residential cleanliness level, and mother’s age at delivery. Sociodemographic factors were assessed using standardized questionnaires administered by trained research staff. Cleanliness and flooring were assessed through visual inspection by study staff based on observed levels of dust accumulation, surface debris, carpet condition, and cleanliness of floors, window sills, and window wells. Our fully adjusted blood lead concentration models included the variables mentioned above as well as child biological sex.

### Statistical analysis

We first calculated descriptive statistics for blood lead concentrations and dust lead loadings, including medians, 25th and 75th percentiles, stratified by covariates. We also estimated proportions of participants reporting firearm ownership, ownership of multiple firearms, storage of unlocked firearms, and storage of ammunition with firearms. In addition, we computed GM blood and dust lead loadings across categories of each firearm-related outcome. To reduce skewness and approximate normality, blood lead concentrations were log_2_-transformed, while dust lead loadings were log_10_-transformed.

We used multivariable linear regression to estimate differences in repeated measures of blood lead levels and dust lead loadings for the four firearm variables after adjusting for covariates. We modeled all available time points jointly using generalized estimating equations (GEE) with a Gaussian family and an exchangeable working correlation structure to account for within-child correlation, fitted with an exposure x categorical time interaction term in each model to assess whether associations varied across follow-up periods. Because interaction tests are underpowered, especially with small to modest sample sizes, we evaluated global heterogeneity of effects across time using a joint Wald test with *p* ≤ 0.2 as a screening threshold for possible time-varying associations.

In sensitivity analyses, we examined whether the associations differed among those participants who remained in the same home for the entire course of follow-up or moved to another location. We did not conduct separate analyses among those who moved given the small sample size of firearm owners within this group (n ~ 10). We examined unadjusted analyses and combined effects where we did not consider exposure x time nteraction terms. We also examined whether firearm ownership differed between new parents and parents who already had one or more children. All analyses were conducted using R [32].

## RESULTS

### Univariate and bivariate associations

Firearm owners (*n* = 59) were more likely to be White (87%), wealthier (50% with $75k or more), college educated (88%) and live in clean (81%) and carpeted (92%) homes compared with non-firearm owners (*n* = 257) (Table 1).

### Associations among all participants (including non-owners)

In unadjusted and adjusted analysis, GM childhood blood lead levels were lower among those living with firearm owners at every time compared with non-firearm owners (Supplementary Table 1). Similarly, dust lead loadings were lower in houses with firearms compared to those without at all three times (Supplementary Table 2). Adjustment for covariates attenuated the magnitude but not direction of these results. A similar pattern of findings was observed comparing non-owners to owners for number of firearms and firearm storage behaviors (Supplementary Table 1, 2). The exceptions were ammunition (GM dust lead loadings were 39% higher [95% CI: −44% to 245%]) and firearm storage behaviors (GM dust lead loadings were 22% higher [95% CI: −36% to 134%]) at baseline in households with firearms, compared to households with no firearms, although these associations were imprecise.

Consistent with the time-specific findings, we observed a similar pattern when we examined all participants at all times (combined together) (Supplementary Table 3). Specifically, we did not observe evidence that the association between firearm exposures and blood lead concentrations differed by timepoint for firearm ownership (interaction *p* = 0.84), number of firearms (interaction *p* = 0.71), ammunition storage (interaction *p* = 0.72), or firearm storage behaviors (interaction *p* = 0.89). This was also observed for dust lead, where there was no difference in the association by time for firearm ownership (interaction *p* = 0.59), number of firearms (interaction *p* = 0.51), ammunition storage (interaction *p* = 0.33), or firearm storage behaviors (interaction *p* = 0.73).

### Associations among firearm owners

In contrast with our findings among all participants, we found positive associations of some firearm behaviors with childhood blood lead levels and dust lead loadings among the participants or households with firearms in both adjusted and unadjusted analysis (Tables 2-4). After covariate adjustment, GM blood lead concentrations were approximately 20% higher at all three time points among children living in a home where firearms were unlocked or not stored (Table 2). Consistent with this, GM dust lead loadings were also higher when firearms were not stored or locked in the household, although the association was weaker at 12 months than baseline or 24 months (Table 3).

In combined models (removal of the exposure x time interaction), results were consistent with time-specific findings (Supplementary Table 4). We did not observe evidence that the association between firearm exposures and blood lead concentrations differed by time. Specifically, we did not observe evidence that the association between firearm exposures and blood lead concentrations differed by number of firearms (interaction *p* = 0.55), ammunition storage (interaction *p* = 0.31), or firearm storage behaviors (interaction *p* = 0.91). The same pattern was observed for dust lead, where there was no difference in the association by timepoint for number of firearms (interaction *p* = 0.57), ammunition storage (interaction *p* = 0.24), or firearm storage behaviors (interaction *p* = 0.83).

### Sensitivity analysis

Restricting analyses to participants who did not move during the first 36 months of follow-up yielded results consistent with, or slightly stronger than, the primary analyses. Among firearm-owning households, children living in homes where firearms were not stored or locked had higher blood lead concentrations across follow-up compared with those in households where firearms were stored. For example, at 12 months, blood lead concentrations were 24.3% higher (95% CI: −2.9%, 58.9%) among children living in households with firearms that were not stored or locked (*n* = 14) compared with those where firearms were stored (*n* = 12). Dust lead loadings showed a comparable pattern: at baseline, households with firearms that were not stored or locked (*n* = 15) had 142.9% higher dust lead loadings (95% CI: 8.7%, 443.0%) than those with stored firearms (*n* = 13). Although confidence intervals were wider due to reduced sample size, the direction and magnitude of associations were consistent with the main findings. Firearm ownership was slightly higher among first-time parents (21.7%, *n* = 36) than parents with prior children (14.7%, *n* = 31) (χ^2^ *p* value = 0.1).

## DISCUSSION

In this prospective pregnancy and birth cohort, we found that firearm and ammunition storage behaviors were related to higher dust lead loadings and childhood blood lead levels among firearm owners. When non-firearm owners were included, firearm ownership was not associated with higher blood lead levels in children age 12–36 months or dust lead loadings in their homes.

To our knowledge, no study has examined children’s risk of lead exposure from household firearms at the individual level. In ecological studies, communities with more firearms had higher rates of elevated child blood lead levels, even when accounting for other occupational sources of lead exposure, lead in paint, water, and aging infrastructure [21-23]. A limitation of these prior studies is that area level firearm rates were used as a proxy for use and the presence of firearms in the home [20].

Prior studies suggest that firearm use and handling may increase the risk of lead exposure in adults. In one study, post-discharge firearm handling with gloves resulted in secondary transfer rates (total gunshot residue [GSR] particles on transferee divided by sum of GSR on shooter) exceeding 81%, even when the firearm was gripped for only 30 s. The half-life of gunshot residue on textiles was 77 minutes, confirming strong surface adhesion [20]. In this same study, those particles could become airborne and resettle onto environmental surfaces, with ambient contamination rates of 41% for vehicles and 32% for clothing. These prior findings underscore the potential for a firearm to act as a localized reservoir of airborne and contact-transferred lead particulates [20]. In the context of our findings, we speculate that storage behaviors, which were related to blood lead concentrations and dust lead loadings among firearm owners, may be a better proxy for frequency of use relative to number of firearms owned, which was not associated with childhood blood lead concentrations or dust lead loadings. We speculate this to be consistent with our theorized pathways (identified via DAG); firearms that are stored unlocked or more readily accessible may be handled more frequently (e.g., for cleaning, transport, or defensive readiness), which could increase opportunities for the transfer of lead-containing gunshot residue to hands, clothing, and household surfaces (Supplementary Fig. 1). However, given the imprecision of dust lead results it is also possible that some differences observed here are the result of our small sample size or imbalances between firearm owners and non-owners (Supplementary Fig. 1).

Indeed, some studies suggest that individuals who own more than one firearm are more likely to cite multiple reasons for ownership (e.g., hunting, recreational shooting, protection), while single firearm owners may report protection as their primary reason [33]. Those who cite protection can be more likely to store at least one firearm loaded and unlocked than those owning for multiple reasons [27, 34]. Other studies found that, among firearm owners who live in States that do not require a permit to carry a firearm, approximately 30% carried handguns in the prior month [35]. These estimates are consistent with data showing that those who are most likely to own more than one firearm are more likely to be white and affluent, and that people who own or collect multiple firearms are more likely to not use the firearms and practice safer storage [33, 36]. Given that firearm use is the most likely exposure mechanism (and remains unmeasurable in our study), it is probable that owning multiple firearms may be less reflective of use when compared to storage-related questions (Supplementary Fig. 1).

National estimates suggest that 40% of households with children own firearms [37]. Of these, 68% are white and college educated or above, 26% live in urban environments, 50% report annual earnings between $25,000 and 99,999, 36% reported storing their firearms unlocked and 37% reported storing their firearms loaded [37]. Other research suggests that, among firearm owners, 66% report owning more than one firearm [38]. Comparatively speaking, our sample has similar demographic characteristics but underreports ownership ( ~ 18%) and is different when stratified by firearm ownership.

Specifically, our sample of firearm owners exhibit different exposure behavior relative to national estimates; our sample reports a greater rate of firearm storage (50% vs. 36%), and lower rate of storing ammunition with firearms (28% vs. 37% who report their firearms stored loaded) and owning multiple firearms (50% vs. 66%). Moreover, our cohort includes a substantial proportion of urban participants. Ohio did not have a child access prevention or safe storage law requiring firearms to be secured in homes with children during the study period. Though firearm laws have loosened in Ohio since the study began, firearm ownership rates have remained stable [39].

The inverse findings in comparing firearm owners to non-owners may be due to several reasons. First, our referent group is more likely to be exposed to residential lead hazards relative to the demographic makeup of our firearm-owners (e.g., more racially diverse, less educated, affluent, and living in less clean environments). This means that those not exposed to firearms in our sample represent a group with higher rates of legacy lead exposure [8]. Second, lead particles from firearms and ammunition may not transfer efficiently to areas that children live and play in. However, this is unlikely because we found that unsafe storage behaviors were associated with higher dust lead loadings and childhood blood lead concentrations among firearm owners.

Lastly, we considered whether firearm ownership differed by number of children in the home, as first time parents may engage in different hygiene behaviors relative to those with more than one child. In our cohort, firearm ownership was only slightly more common among first-time parents. Moreover, because firearm ownership was assessed during pregnancy and not reassessed over follow-up and given the pediatric risks inherent in household firearm ownership, it is plausible that divestment may be more likely to occur among first-time parents [40]. In this case, there would be non-differential exposure misclassification, biasing associations toward the null rather than being responsible for any protective effects.

Our study had several limitations. First, the number of firearm-owning households in our cohort was small (*n* ~ 55), which limited our statistical power. However, the use of a prospective birth cohort design with repeated lead measurements allowed us to temporally sequence our firearm use metrics and lead exposure measures. In addition, we were able to leverage repeated measures across three time points, which increased power and precision. Second, our sample is not representative of firearm-owning households in the U.S. Third, all firearm exposure variables were based on maternal self-report during pregnancy, rather than direct observation or verification. However, maternal reporting during pregnancy may reduce recall bias and may increase accuracy, as mothers may be particularly attuned to household safety and non-differential underreporting unsafe practices would attenuate observed associations. Relatedly, we were also unable to directly measure firearm use. While unsafe storage may be a proxy for use or handling firearms, this is not definitive and thus, we cannot determine whether the observed associations were due to recent discharges, residue on clothing, or long-term lead shedding from stored firearms. Yet prior experimental evidence shows that firearm-related lead is transferable and persistent even with minimal handling, and self-report of firearm ownership and storage behaviors are commonly used in epidemiologic studies of firearm exposure [20, 27, 41]. Finally, we only assessed firearm ownership and storage behaviors once during pregnancy. It is possible that caregivers purchase or divest of firearms, change their storage behavior, or otherwise adjust firearm safety habits after having a child. Although there is limited literature on these changes, such exposure misclassification would likely be non-differential with respect to later lead measurements; this bias would be likely to attenuate our findings to the null.

This study is the first to examine the relationship between household firearm storage behaviors and pediatric lead exposure at the individual level using prospectively collected dust lead loadings and blood lead concentrations. Among firearm owners, we found that unsafe firearm and ammunition storage were associated with elevated household dust lead and higher child blood lead levels. These findings suggest that firearms represent a source of pediatric lead exposure. As firearm ownership is widespread, further research is needed to quantify the broader population-level burden of firearm-related lead exposure.

### Public health implications

In the U.S., 40% of adults with children (representing nearly 30 million children) report living in a household with a firearm [37]. Despite major reductions in lead exposures, elevated pediatric blood lead levels remain common, and household firearms are rarely considered within existing lead prevention frameworks [14]. Our findings suggest that firearm and ammunition storage behaviors may contribute to residential dust lead contamination and elevated blood lead levels among children living in firearm-owning households, representing a potentially modifiable exposure pathway that can be addressed within existing lead prevention and anticipatory guidance frameworks. When firearm use is present or self-identified, clinicians may offer brief, nonjudgmental, and informational counseling—analogous to occupational take-home lead guidance—focused on storage practices, hygiene, and environmental contamination, without requiring changes to clinical workflows or documentation.

## Supplementary Material

Supplemental Tables and Figures_Clean

**Supplementary information** The online version contains supplementary material available at https://doi.org/10.1038/s41370-026-00928-w.

## Figures and Tables

**Table 1.** Total sample size, complete-covariate median blood lead concentrations and dust lead loadings, and prevalence of complete-covariate self-reported firearm ownership, number of firearms, and firearm and ammunition storage behavior among The HOME Study (*n* = 420).

Covariate	Total Study N	Median baseline dust lead μg/ft^2^ (25th, 75th)	Median 12 month dust lead μg/ft^2^ (25th, 75th)	Median 24 month dust lead μg/ft^2^ (25th, 75th)	Median 12 month blood lead concentration, μg/L (25th, 75th)	Median 24 month blood lead concentration, μg/L (25th, 75^th^)	Median 36 month blood lead concentration, μg/L (25th, 75th)	Firearm Owners, *n* (%)	Multiple Firearm Ownership, *n* (%)	Firearms Unlocked/ Not Stored, *n* (%)	Ammunition Stored Unlocked, *n* (%)
Mother’s Income											
$2.5k-17.5k	88	3.0 (1.0,6.3)	2.2 (1.3, 10.2)	1.7 (0.6, 4.5)	21.3 (16.5, 28.4)	19.5 (15.1, 25.1)	17.5 (14.1, 22.5)	1 (1.1)	1 (1.1)	1 (1.1)	1 (1.1)
$22.5k-35k	75	2.5 (1.0,6.0)	1.7 (0.6, 5.4)	1.0 (0.4, 4.3)	16.1 (12.2, 20.6)	14.7 (11.1, 18.5)	13.2 (10.2, 16.9)	8 (12.7)	3 (4.8)	3 (4.8)	0 (0.0)
$45k-65k	109	1.4 (0.7,3.5)	1.1 (0.5, 3.0)	1.0 (0.4, 3.0)	13.6 (11.2, 16.6)	12.5 (10.2, 15.0)	11.4 (9.2, 13.6)	22 (25.3)	13 (14.9)	6 (6.9)	4 (4.6)
$75k-95k	82	0.9 (0.4,4.0)	1.1 (0.5, 2.8)	0.8 (0.4, 2.9)	12.4 (10.3, 15.1)	11.3 (10.2, 15.0)	10.5 (8.8, 12.7)	20 (25.0)	10 (12.5)	12 (15.0)	8 (10.0)
$105k-155k	66	1.6 (0.6,3.3)	0.9 (0.4, 2.4)	1.1 (0.5, 2.0)	12.4 (10.8, 14.6)	11.4 (10.0, 13.3)	10.6 (9.3, 12.1)	16 (26.2)	5 (8.2)	9 (14.8)	5 (8.2)
Mother’s Age at Delivery											
<25 Years	102	2.4 (1.1,6.2)	2.4 (1.0, 7.2)	1.5 (0.6, 5.0)	17.4 (13.7, 24.6)	15.8 (12.6, 21.6)	14.5 (11.4, 19.5)	9 (9.7)	4 (4.3)	4 (4.3)	1 (1.1)
25-29 Years	123	1.3 (0.6,4.5)	1.4 (0.5, 3.6)	1.2 (0.5, 3.0)	14.3 (11.3, 19.4)	13.0 (10.2, 17.5)	11.9 (9.2, 16.2)	20 (18.9)	11 (10.4)	6 (5.7)	7 (6.6)
30-34 Years	131	1.2 (0.5,3.5)	1.1 (0.4, 2.7)	0.8 (0.4, 3.1)	13.0 (10.9, 16.4)	11.8 (9.9, 14.7)	10.8 (9.4, 13.2)	20 (16.8)	8 (6.7)	13 (10.9)	4 (3.4)
35+ Years	64	2.1 (0.8,6.2)	1.2 (0.6, 2.9)	0.8 (0.5, 2.0)	13.6 (11.1, 17.2)	12.5 (10.6, 16.1)	11.5 (9.8, 14.2)	18 (29.5)	9 (14.8)	8 (13.1)	6 (9.8)
Mother’s Education											
College or Above	306	1.6 (0.6,4.7)	1.2 (0.5, 3.3)	0.9 (0.4, 2.6)	13.1 (10.9, 16.7)	11.9 (10.0, 15.0)	10.9 (9.2, 13.6)	58 (20.7)	28 (10.0)	26 (9.3)	16 (5.7)
High School or Less	100	1.9 (0.8,5.0)	1.9 (0.6, 6.9)	2.1 (0.7, 6.4)	20.9 (15.8, 27.5)	18.5 (14.5, 24.7)	17.3 (13.1, 22.1)	9 (9.1)	4 (4.0)	5 (5.1)	2 (2.0)
Mother’s Race											
White	257	1.2 (0.5,4.8)	1.1 (0.5, 2.9)	0.8 (0.4, 2.3)	12.8 (10.8, 16.2)	11.8 (9.9, 14.6)	10.8 (9.1, 13.2)	56 (23.9)	27 (11.5)	27 (11.5)	14 (6.0)
Non-White	158	2.2 (0.9,5.1)	1.9 (0.8, 7.2)	1.9 (0.8, 4.7)	18.9 (15.0, 25.3)	17.4 (13.5, 22.6)	15.9 (12.4, 19.9)	11 (7.6)	5 (3.5)	4 (2.8)	4 (2.8)
Cleanliness Level											
Appears Clean	238	1.2 (0.5,3.4)	0.9 (0.4, 2.3)	0.8 (0.4, 1.9)	12.7 (10.7, 16.2)	11.7 (9.9, 14.7)	10.7 (9.1, 13.3)	54 (23.9)	24 (10.6)	24 (10.6)	15 (6.6)
Does not appear clean	157	3.1 (1.1,7.5)	2.6 (1.1, 7.3)	1.7 (0.7, 5.6)	17.5 (13.7, 24.6)	15.8 (12.2, 21.7)	14.2 (11.2, 19.5)	13 (8.6)	8 (5.3)	7 (4.6)	3 (2.0)
Flooring Level											
Carpeted - High	120	1.4 (0.6, 5.4)	1.5 (0.7, 4.8)	1.0 (0.6, 2.4)	13.5 (11.1, 15.5)	12.2 (10.3, 13.8)	12.2 (9.6, 16.7)	20 (17.2)	11 (9.5)	10 (8.6)	6 (5.2)
Carpeted - Medium	190	1.2 (0.5, 3.5)	1.2 (0.5, 2.9)	0.8 (0.4, 3.1)	13.9 (11.4, 18.7)	12.7 (10.5, 17.0)	11.5 (9.6, 14.0)	36 (19.6)	14 (7.6)	17 (9.2)	9 (4.9)
Carpeted - Low	17	1.9 (0.9, 5.3)	1.6 (0.5, 2.4)	1.7 (0.8, 3.8)	14.6 (11.1, 20.4)	13.4 (10.4, 18.2)	11.1 (9.5, 12.4)	5 (29.4)	2 (11.8)	2 (11.8)	0 (0.0)	
Not Carpeted	62	3.5 (1.2, 8.1)	1.3 (0.5, 5.1)	1.6 (0.9, 6.1)	14.7 (12.4, 19.8)	13.4 (11.3, 17.8)	12.3 (10.7, 16.3)	6 (10.2)	5 (8.5)	2 (3.4)	3 (5.1)	
Move Status											
Did not Move	252	1.3 (0.5, 4.3)	1.1 (0.5, 2.6)	0.9 (0.5, 3.0)	13.1 (10.6, 16.4)	11.9 (9.8, 14.9)	10.9 (9.0, 13.6)	50 (22.8)	22 (10.0)	23 (10.5)	10 (4.6)	
Moved by Month 12	69	2.5 (0.8, 5.9)	1.1 (0.5, 5.8)	1.2 (0.6, 5.7)	17.2 (12.6, 22.7)	15.5 (11.6, 19.9)	14.2 (10.7, 17.4)	8 (12.9)	6 (9.7)	3 (4.8)	4 (6.5)	
Moved by Month 24	40	1.7 (0.5, 6.4)	2.6 (1.0, 4.7)	1.0 (0.4, 3.8)	14.0 (11.6, 21.3)	12.8 (10.5, 19.2)	11.5 (9.5, 17.3)	4 (10.0)	2 (5.0)	3 (7.5)	3 (7.5)	
Moved by Month 36	20	2.1 (1.3, 7.9)	2.1 (1.2, 4.1)	1.7 (1.0, 3.6)	15.8 (13.0, 19.3)	14.3 (11.6, 17.1)	12.9 (10.7, 15.2)	5 (25.0)	2 (10.0)	2 (10.0)	1 (5.0)	
Moved more than once	39	2.5 (1.0, 4.8)	1.7 (0.7, 4.3)	0.7 (0.5, 1.9)	17.8 (15.3, 27.3)	16.9 (13.5, 24.2)	14.6 (12.0, 21.1)	0 (0.0)	0 (0.0)	0 (0.0)	0 (0.0)	
Prior Children											
No prior children	178	1.8 (0.7, 5.1)	1.3 (0.5, 3.9)	1.0 (0.4, 3.1)	13.7 (11.3, 17.1)	12.5 (10.3, 15.2)	11.4 (9.5, 13.9)	36 (21.7)	16 (9.6)	18 (10.8)	10 (6.0)	
1 or more prior children	227	1.7 (0.6, 4.8)	1.4 (0.6, 3.5)	1.0 (0.5, 3.3)	14.7 (11.6, 22.1)	13.5 (10.7, 19.8)	12.4 (9.9, 17.6)	31 (14.7)	16 (7.6)	13 (6.2)	8 (3.8)	

**Table 2.** Adjusted^a^ percent differences in blood lead concentrations (μg/L) among firearm owners, by number of firearms, ammunition storage and firearm storage behaviors (The HOME Study).

Exposure, Time		12 Month			24 Month			36 Month		
		*n*	Unadjusted GM^b^ Blood Lead	Adjusted % Difference (95% CI^c^)	*n*	Unadjusted GM^b^ Blood Lead	Adjusted % Difference (95% CI)	*n*	Unadjusted GM^b^ Blood Lead	Adjusted % Difference (95% CI)
Number of Firearms	1	26	12.9	—	24	12.2	—	17	10.6	—
	2+	29	13.4	2.4% (−16.0%, 25.0%)	24	11.8	1.1% (−16.6%, 22.6%)	19	10.5	0.1% (−17.1%, 20.9%)
Ammunition storage behaviors	Stored separately	35	12.3	—	32	11.4	—	22	9.5	—
	Not stored separately	17	13.5	9.2% (−12.2%, 35.9%)	13	11.8	8.0% (−12.4%, 33.1%)	11	11.1	6.3% (−13.1%, 29.9%)
Firearm storage behaviors	All stored/locked	27	11.9	—	20	11.0	—	15	9.3	—
	Not all stored/locked	27	14.2	19.4% (−0.2%, 42.8%)	27	12.5	20.2% (0.9%, 43.2%)	21	11.4	21.3% (2.6%, 43.4%)

a Adjusted for the natural spline terms (3) for income ($2.5k–17.5k, $22.5k–35k, $45k–65k, $75k–95k, $105k–155k), race/ethnicity (non-Hispanic Black, White & Other), carpet level (high, medium, low, no carpet), cleanliness level (appears clean, does not appear clean), mother's age at delivery, and child biological sex.

b GM=Geometric Mean.

c CI: Confidence Interval.

**Table 3.** Adjusted^a^ percent differences in dust loadings (μg/ft^2^) among firearm owners, by number of firearms, ammunition storage and firearm storage behaviors (The HOME Study).

Exposure, Time		Baseline			12 Month			24 Month		
		*n*	Unadjusted GM^b^ Dust Lead	Adjusted % Difference (95% CI^c^)	*n*	Unadjusted GM^b^ Dust Lead	Adjusted % Difference (CI)	*n*	Unadjusted GM^b^ Dust Lead	Adjusted % Difference (CI)
Number of Firearms	1	28	1.2	—	25	1.1	—	25	0.9	—	
	2+	31	1.3	−7.0% (−57.2%, 102.2%)	30	0.8	−39.7% (−69.0%, 17.1%)	25	0.8	−4.9% (−51.1%, 84.9%)	
Ammunition Storage Behaviors	Stored separately	40	0.9	—	35	1.0	—	33	1.0	—	
	Not stored separately	17	1.9	76.3% (−33.3%, 366.3%)	17	0.7	−33.4% (−71.2%, 53.8%)	14	0.6	−32.7% (−65.3%, 30.4%)	
Firearm Storage behaviors	All stored/locked	29	0.9	—	28	0.9	—	21	0.7	—	
	Not all stored/locked	30	1.6	69.4% (−16.6%, 244.1%)	27	0.9	25.6% (−39.5%, 160.5%)	28	0.9	35.7% (−29.0%, 159.4%)	

a Adjusted for the natural spline terms (3) for income ($2.5k–17.5k, $22.5k–35k, $45k–65k, $75k–95k, $105k–155k), race/ethnicity (non-Hispanic Black, White & Other), carpet level (high, medium, low, no carpet), cleanliness level (appears clean, does not appear clean), and mother’s age at delivery

b GM=Geometric Mean.

c CI: Confidence Interval.

**Table 4.** Unadjusted percent differences in blood lead concentrations (μg/L) and dust lead loadings (μg/ft^2^) among firearm owners, by number of firearms, ammunition storage and firearm storage behaviors (the HOME Study).

Exposure, Outcome		Blood Lead Concentrations (95% CI^a^)				Dust lead concentrations (95% CI)			
		*n*	12 Month	24 Month	36 Month	*n*	Baseline	12 Month	24 Month
Number of Firearms	1	27	—	—	—	28	—	—	—
	2+	30	3.2% (−13.7%, 23.2%)	2.4% (−13.6%, 21.3%)	1.3% (−13.8%, 19.2%)	31	7.1% (−47.9%, 120.1%)	−31.7% (−67.1%, 41.9%)	−9.1% (−54.8%, 82.5%)
Ammunition Storage Behaviors	Stored separately	38	—	—	—	40	—	—	—
	Not stored separately	17	9.8% (−12.2%, 37.3%)	8.9% (−11.9%, 34.6%)	6.8% (−12.8%, 30.6%)	17	113.7% (−15.7%, 441.4%)	−33.0% (−73.4%, 68.6%)	−39.0% (−68.1%, 16.7%)
Firearm Storage Behaviors	All stored/locked	27	—	—	—	29	—	—	—
	Not all stored/locked	30	18.2% (−0.4%, 40.4%)	18.5% (0.8%, 39.3%)	18.4% (1.5%, 38.1%)	30	81.6% (−9.8%, 265.9%)	9.6% (−47.9%, 130.2%)	28.1% (−34.9%, 152.0%)

a CI: Confidence Interval

## Data Availability

The data that support the findings of this study are derived from the Health Outcomes and Measures of the Environment (HOME) Study and contain sensitive participant information. Restrictions apply to the availability of these data under the study’s data use agreements and institutional review board approvals. Requests for de-identified data may be made to HOME Study investigators and may be rendered with appropriate approvals.
