# Supplementary material for: Firearms, storage practices, and child blood lead levels and household dust lead loadings: findings from the HOME study
Source: J Expo Sci Environ Epidemiol. Author manuscript; Available in PMC 2026 Nov 1. (PMC13723769; doi:10.1038/s41370-026-00928-w)
Supplement: Supplementary file 1 [file NIHMS2215301-supplement-Supplemental_Tables_and_Figures_Clean.docx]

**Supplemental Table 1. Adjusted^a^ percent differences in blood lead concentration (µg/L) by firearm ownership, number of firearms, ammunition storage and firearm storage behaviors (The HOME Study).**

| Exposure, Outcome | | Month 12 | | | Month 24 | | | Month 36 | | |
| --- | --- | --- | --- | --- | --- | --- | --- | --- | --- | --- |
|  |  | n | Unadjusted GM^b^ Blood Lead | Adjusted % Difference  (95% CI^c^) | n | Unadjusted GM Blood Lead | Adjusted % Difference  (95% CI) | n | Unadjusted GM Blood Lead | Adjusted % Difference  (95% CI) |
| Firearm Ownership | No | 257 | 15.5 | -- | 220 | 13.8 | -- | 195 | 12.4 | -- |
|  | Yes | 56 | 13.1 | -7.9%  (-16.9%, 2.0%) | 49 | 12.0 | -7.6%  (-16.2%, 1.8%) | 37 | 10.6 | -7.3%  (-15.6%, 1.8%) |
| Number of Firearms | 0 | 257 | 15.5 | -- | 220 | 13.8 | -- | 195 | 12.4 | -- |
|  | 1 | 26 | 12.9 | -7.8%  (-21.0%, 7.6%) | 24 | 12.2 | -7.0%  (-19.9%, 8.0%) | 17 | 10.6 | -6.2%  (-19.0%, 8.5%) |
|  | 2+ | 29 | 13.4 | -7.5%  (-18.7%, 5.0%) | 24 | 11.8 | -7.9%  (-18.2%, 3.7%) | 19 | 10.5 | -7.9%  (-17.6%, 2.9%) |
| Ammunition Storage behaviors | No Firearms | 257 | 15.5 | -- | 220 | 13.8 | -- | 195 | 12.4 | -- |
|  | Stored separately | 34 | 12.3 | -13.2%  (-21.5%, -4.0%) | 31 | 11.4 | -12.6%  (-20.6%, -3.7%) | 21 | 9.5 | -11.7%  (-19.6%, -3.0%) |
|  | Not stored separately | 17 | 13.5 | -6.2%  (-24.5%, 16.6%) | 13 | 11.8 | -6.6%  (-23.6%, 14.3%) | 11 | 11.1 | -6.9%  (-23.3%, 12.9%) |
| Firearm Storage behaviors | No Firearms | 257 | 15.5 | -- | 220 | 13.8 | -- | 195 | 12.4 | -- |
|  | All  stored/locked | 26 | 11.9 | -15.1%  (-26.7%, -1.6%) | 20 | 11.0 | -14.8%  (-26.0%, -1.8%) | 15 | 9.3 | -14.6%  (-25.6%, -2.1%) |
|  | Not all stored/locked | 27 | 14.2 | -1.3%  (-13.8%, 13.1%) | 26 | 12.5 | -1.3%  (-13.0%, 11.9%) | 20 | 11.5 | -1.0%  (-12.0%, 11.4%) |

^a^Adjusted for the natural spline terms (3) for income ($2.5k-17.5k, $22.5k-35k, $45k-65k, $75k-95k, $105k-155k), race/ethnicity (non-Hispanic Black, White & Other), carpet level (high, medium, low, no carpet), cleanliness level (appears clean, does not appear clean), mother’s age at delivery, and child biological sex

^b^GM=Geometric Mean

^c^CI: Confidence Interval

**Supplemental Table 2. Adjusted^a^ percent differences in dust lead concentration (µg/ft²) by firearm ownership, number of firearms, ammunition storage and firearm storage behaviors (The HOME Study).**

| Exposure, Outcome | | Baseline | | | Month 12 | | | Month 24 | | |
| --- | --- | --- | --- | --- | --- | --- | --- | --- | --- | --- |
|  |  | n | Unadjusted GM Dust Lead | Adjusted % Difference, (95% CI^c^) | n | Unadjusted GM Dust Lead | Adjusted % Difference, (95% CI) | n | Unadjusted GM Dust Lead | Adjusted % Difference, (95% CI) |
| Firearm Ownership | No | 256 | 1.9 | -- | 238 | 1.6 | -- | 219 | 1.3 | -- |
|  | Yes | 61 | 1.2 | -11.3%  (-41.6%, 34.9%) | 56 | 0.9 | -28.2%  (-51.3%, 5.8%) | 51 | 0.9 | -14.3%  (-41.1%, 24.7%) |
|  | 0 | 256 | 1.9 | -- | 238 | 1.6 | -- | 219 | 1.3 | -- |
| Number of Firearms | 1 | 28 | 1.2 | -0.5%  (-15.2%, 16.8%) | 25 | 1.1 | -1.7%  (-16.1%, 15.2%) | 25 | 0.9 | -4.8%  (-19.8%, 13.0%) |
|  | 2+ | 31 | 1.3 | -4.9%  (-20.7%, 14.0%) | 30 | 0.8 | -15.2%  (-27.2%, -1.4%) | 25 | 0.8 | -4.3%  (-16.1%, 9.2%) |
| Ammunition Storage behaviors | No Firearms | 256 | 1.9 | -- | 238 | 1.6 | -- | 219 | 1.3 | -- |
|  | Stored separately | 40 | 0.9 | -30.6%  (-54.2%, 5.2%) | 35 | 1.0 | -20.9%  (-47.1%, 18.1%) | 33 | 1.0 | -6.7%  (-42.5%, 51.3%) |
|  | Not stored separately | 17 | 1.9 | 38.8%  (-44.2%, 245.2%) | 17 | 0.7 | -43.2%  (-74.9%, 28.8%) | 14 | 0.6 | -32.9%  (-58.4%, 8.4%) |
| Firearm Storage behaviors | No Firearms | 256 | 1.9 | -- | 238 | 1.6 | -- | 219 | 1.3 | -- |
|  | All stored/locked | 29 | 0.9 | -34.6%  (-57.6%, 0.9%) | 28 | 0.9 | -33.6%  (-55.9%, -0.1%) | 21 | 0.7 | -24.9%  (-51.2%, 15.5%) |
|  | Not all stored/locked | 30 | 1.6 | 22.0%  (-36.4%, 134.1%) | 27 | 0.9 | -22.8%  (-59.0%, 45.1%) | 28 | 0.9 | -7.5%  (-46.7%, 60.4%) |

^a^Adjusted for the natural spline terms (3) for income ($2.5k-17.5k, $22.5k-35k, $45k-65k, $75k-95k, $105k-155k), race/ethnicity (non-Hispanic Black, White & Other), carpet level (high, medium, low, no carpet), cleanliness level (appears clean, does not appear clean), and mother’s age at delivery.

^b^GM=Geometric Mean

^c^CI: Confidence Interval

**Supplemental Table 3. Unadjusted and adjusted^a^ percent differences in combined (at 12 and 24 months) blood lead concentrations (µg/L) and dust lead loadings (µg/ft^2^) by firearm ownership, number of firearms, and household availability of firearms (The HOME Study).**

| Exposure, Outcome | | Blood Lead Concentrations | | | | Dust Lead Concentrations | | | |
| --- | --- | --- | --- | --- | --- | --- | --- | --- | --- |
|  |  | n | Unadjusted GM Blood Lead | Unadjusted % Difference, (95% CI^c^) | Adjusted % Difference, (95% CI) | n | Unadjusted GM Dust Lead | Unadjusted % Difference, (95% CI) | Adjusted % Difference, (95% CI) |
| Firearm Ownership | No | 264 | 15.5 | -- | -- | 262 | 1.6 | -- | -- |
|  | Yes | 59 | 13.1 | -15.2%  (-23.0%, -6.6%) | -5.11%  (-13.7%, 4.3%) | 61 | 1.0 | -38.5%  (-51.3%, -22.4%) | -16.7%  (-34.3%, 5.7%) |
| Number of Firearms | 0 | 264 | 15.5 | -- | -- | 262 | 1.6 | -- | -- |
|  | 1 | 27 | 12.9 | -16.2%  (-26.5%, -4.4%) | -4.9%  (-17.7%, 9.9%) | 28 | 1.1 | -33.5%  (-51.7%, -8.35%) | -5.7%  (-32.0%, 30.6%) |
|  | 2+ | 30 | 13.4 | -14.0%  (-24.6%, -2.0%) | -4.9%  (-15.4%, 6.9%) | 31 | 0.9 | -41.6%  (-57.3%, -20.0%) | -24.6%  (-44.9%, 3.25%) |
| Ammunition Storage Behaviors | No Firearms | 264 | 14.0 | -- | -- | 262 | 1.6 | -- | -- |
|  | Stored separately | 38 | 11.2 | -19.9%  (-27.0%, -12.0%) | -10.0%  (-18.0%, -1.1%) | 40 | 1.0 | -39.7%  (-53.4%, -22.1%) | -19.0%  (-37.4%, 5.0%) |
|  | Not stored separately | 17 | 12.3 | -12.5%  (-28.9%, 7.7%) | -3.5%  (-20.6%, 17.3%) | 17 | 1.0 | -40.6%  (-63.3%, -3.8%) | -17.8%  (-49.0%, 32.3%) |
| Firearm Storage behaviors | No Firearms | 264 | 14.0 | -- | -- | 262 | 1.6 | -- | -- |
|  | All stored/locked | 27 | 10.9 | -22.3%  (-31.8%, -11.6%) | -11.3%  (-22.5%, 1.6%) | 29 | 0.8 | -48.4%  (-60.2%, -33.0%) | -30.4%  (-46.1%, -10.1%) |
|  | Not all stored/locked | 30 | 12.8 | -9.9%  (-20.5%, 2.2%) | -0.1%  (-11.4%, 12.7%) | 30 | 1.1 | -29.6%  (-50.7%, 0.4%) | -2.9%  (-32.6%, 39.8%) |

^a^Adjusted for the natural spline terms (3) for income ($2.5k-17.5k, $22.5k-35k, $45k-65k, $75k-95k, $105k-155k), race/ethnicity (non-Hispanic Black, White & Other), carpet level (high, medium, low, no carpet), cleanliness level (appears clean, does not appear clean), and mother’s age at delivery. Blood lead models are additionally adjusted for child biological sex.

^b^GM=Geometric Mean

^c^CI: Confidence Interval

**Supplemental Table 4. Unadjusted and adjusted^a^ percent differences in combined (at 12 and 24 months) blood lead concentrations (µg/L) and dust lead loadings (µg/ft^2^) among firearm owners, by number of firearms, and household availability of firearms (The HOME Study).**

| Exposure, Outcome | | Blood Lead Concentrations | | | | Dust Lead Concentrations | | | |
| --- | --- | --- | --- | --- | --- | --- | --- | --- | --- |
|  |  | n | Unadjusted GM Blood Lead | Unadjusted % Difference, (95% CI^c^) | Adjusted % Difference, (95% CI) | n | Unadjusted GM Dust Lead | Unadjusted % Difference, (95% CI) | Adjusted % Difference, (95% CI) |
| Number of Firearms | 1 | 27 | 11.2 | -- | -- | 27 | 1.3 | -- | -- |
|  | 2+ | 30 | 11.2 | 2.7%  (-13.7%, 22.2%) | 2.3%  (-14.5%, 22.3%) | 30 | 1.1 | -10.4%  (-39.5%, 32.6%) | -18.2%  (-44.9%, 21.3%) |
| Ammunition Storage Behaviors | Stored separately | 38 | 11.2 | -- | -- | 39 | 1.1 | -- | -- |
|  | Not stored separately | 17 | 12.3 | 9.2%  (-12.2%, 35.8%) | 8.49%  (-10.5%, 35.8%) | 16 | 1.2 | 6.4%  (-34.6%, 73.1%) | -1.6%  (-38.4%, 57.0%) |
| Firearm Storage Behaviors | All stored/locked | 27 | 10.9 | -- | -- | 28 | 0.9 | -- | -- |
|  | Not all stored/locked | 30 | 12.8 | 16.1%  (-1.9%, 37.33%) | 14.8%  (-2.3%, 35.0%) | 29 | 1.4 | 48.3%  (1.1%, 117.6%) | 55.1%  (7.1%, 124.6%) |

^a^Adjusted for the natural spline terms (3) for income ($2.5k-17.5k, $22.5k-35k, $45k-65k, $75k-95k, $105k-155k), race/ethnicity (non-Hispanic Black, White & Other), carpet level (high, medium, low, no carpet), cleanliness level (appears clean, does not appear clean), and mother’s age at delivery. Blood lead models are additionally adjusted for child biological sex.

^b^GM=Geometric Mean

^c^CI: Confidence Interval

**Supplemental Figure 1.** Directed Acyclic Graph (DAG) of the association between firearm ownership, storage behaviors, and number of firearms with house dust and child blood lead levels (The HOME Study).

Green nodes are exposures. Transparent grey nodes are unobserved variables. White nodes are adjusted covariates. Blue nodes are outcomes.
